# Development and internal validation of a prescriptive multi-task learning model for horizontal strabismus surgery planning

**DOI:** 10.1186/s12886-026-04628-9

**Published:** 2026-01-21

**Authors:** Jieyue Wang, Xiaoying Wu, Sheng Ou

**Affiliations:** 1 Eye Center of Xiangya Hospital, Central South University, 87 Xiangya Road, Changsha, Hunan Province 410008 China; 2 Hunan Key Laboratory of Ophthalmology, Central South University, Changsha, Hunan Province 410008 China; 3 National Clinical Research Center for Geriatric Disorders, Xiangya Hospital, Central South University, Changsha, Hunan Province 410008 China

**Keywords:** Strabismus, Machine learning, Prescriptive model, Personalised surgery, Deep learning, Clinical decision support

## Abstract

**Background & aim:**

Horizontal strabismus affects ≈ 1.9% of the global population. Traditional “1 mm ≈ 2 Δ” nomograms disregard patient heterogeneity, leaving re-operation rates at 7–8% even after primary horizontal surgery. We aimed to develop a single prescriptive model that simultaneously predicts which horizontal extra-ocular muscles require surgery and the precise recession/resection dose for each, following the TRIPOD + AI reporting checklist.

**Methods:**

In this retrospective single-centre study, 634 consecutive patients (2019–2024) undergoing primary horizontal-muscle surgery were analysed. Fourteen routinely recorded pre-operative variables—including age, prism-cover deviation, axial-length metrics, refractive error and visual acuity—fed a fully connected multi-task neural network with a shared trunk and two heads: (i) 8-label classification for muscle-procedure selection and (ii) 8-output regression for surgical dose. Model development exceeded recommended sample-size heuristics for an expected AUC ≥ 0.90 and was internally validated with multilabel-stratified 10-fold cross-validation.

**Results:**

The model achieved excellent discrimination for muscle selection (macro-AUC 0.97 ± 0.01; macro-MCC 0.83) with near-perfect calibration (ECE 0.008). Dose predictions were highly accurate (MAE 0.42 ± 0.04 mm; RMSE 0.54 ± 0.07 mm; R² 0.86 ± 0.04); 95% of estimates lay within ± 0.30 mm of the surgeon’s plan. Exact match of the entire surgical plan reached 55%, far surpassing the majority baseline of 17%. These figures markedly outperform earlier regression-only approaches that reported MAE 0.5–0.8 mm and indication-level AUC 0.82.

**Conclusions:**

A transparent multi-task learning model can replicate expert, patient-specific surgical plans for horizontal strabismus with sub-millimetre precision. The tool could standardise planning and reduce inter-surgeon variability; multi-centre external validation remains essential.

**Supplementary Information:**

The online version contains supplementary material available at 10.1186/s12886-026-04628-9.

## Strengths and limitations of this study

Strengths of this study

Limitations of this study

- First integrated prescriptive model that jointly predicts which horizontal extra-ocular muscles to operate and their recession/resection dose, outperforming earlier regression-only SVM and decision-tree approaches (0.97 macro-AUC vs. 0.82; 0.42 mm MAE vs. 0.5–0.8 mm).
- Robust, adequately powered dataset of 634 consecutive cases; exceeds published sample-size guidance for developing an AUC ≥ 0.90 model and maintains a favourable events-per-parameter ratio.
- Methodological rigour: multilabel-stratified 10-fold cross-validation, bootstrap CIs, and probability calibration executed in line with TRIPOD + AI reporting standards, enhancing transparency and reproducibility.
- Ground-truth surgical plans were associated with uniformly positive outcomes, ensuring that the model learned from high-quality examples.
- High clinical fidelity: 55% exact plan match and 95% of dose errors ≤ ± 0.30 mm—well below the 0.5 mm threshold deemed clinically perceptible—suggest potential to standardise surgical planning.

- Single-centre, retrospective design may encode local practice patterns; optimism bias is possible without external validation in diverse settings.
- Class imbalance effects: rare procedures (e.g., left lateral-rectus resection) have fewer training examples and slightly lower accuracy despite stratified sampling.

**Supplementary Information:**

The online version contains supplementary material available at 10.1186/s12886-026-04628-9.

## Introduction

Horizontal strabismus, defined as a persistent ocular misalignment in the horizontal plane, affects ≈ 1.9% of the global population [1]. Such misalignment compromises binocular vision, diminishes quality of life and carries psychosocial stigma. When deviation angles exceed clinical thresholds, the Adult Strabismus Preferred Practice Pattern^®^ (AAO) recommends recession or resection of the medial or lateral rectus muscles [2]. Precise dosing of these procedures is therefore critical for surgical success.

For more than a century surgeons have relied on the rule that *1 mm of muscle displacement ≈ 2 prism-dioptres*, a nomogram that ignores patient-specific factors such as age, axial length and anisometropia [3]. A recent dose–effect model explained only 78% of postoperative variance despite detailed measurement of recession/resection length [4]. Large national audits still report horizontal-strabismus re-operation rates of 7–8% [5], underscoring the need for more reliable, data-driven planning tools.

Paragraph 3 – Prior Work.

Machine-learning research to date has focused exclusively on dose prediction. *De Almeida et al.* applied support-vector regression to 114 cases, yielding dose errors of 0.5–0.8 mm and an indication-level AUC of 0.82 [6]. *Leite et al.* used a multiple-output regression tree in 153 patients but reported lateral-rectus dose R² < 0.70 with systematic overestimation [7]. Crucially, these systems do not decide which muscles should be operated.

To address this gap we developed and internally validated a multi-task neural network that ingests routine pre-operative data and outputs (i) binary recommendations for each of eight muscle-procedure options and (ii) personalised recession/resection doses. The investigation follows the TRIPOD + AI reporting checklist [8] and conforms to the STROBE guideline for observational studies [9].

We detail the 634-patient cohort, modelling pipeline and 10-fold stratified cross-validation results; sample-size adequacy was confirmed against contemporary prediction-research guidance [10].

## Materials and methods

### Study design and setting

We performed a retrospective cohort study at the Eye Center of Xiangya Hospital, a tertiary referral centre that performs > 5 000 strabismus procedures annually. The study window spanned 1 March 2021 to 29 March 2024, capturing every primary horizontal-muscle operation logged in the electronic medical record (EMR) and the surgeon-maintained strabismus registry. The protocol was approved by the Xiangya Hospital Clinical Medical Ethics Review Committee (Approval No. 2025040585, 9 Apr 2025); written informed consent was waived because only de-identified data were analysed. The design and reporting adhere to TRIPOD-AI [7] and STROBE guidelines [8].

### Participants

A total of 634 consecutive patients were identified. To ensure high-quality and consistent ground-truth labels, all surgical plans were formulated by a fixed expert team led by the corresponding author, a committee member of the Chinese Association of Strabismus & Pediatric Ophthalmology and Vice-Head of the Hunan Strabismus Group. This team performs over 500 strabismus procedures annually.

While immediate post-operative alignment was consistently recorded, complete long-term functional outcome data were not systematically available for model training. As a tertiary referral centre, a significant proportion of our patients travel from distant provinces. Despite established follow-up protocols, the complete 1-month return rate is approximately 20%, often due to economic constraints or patients discontinuing care once cosmetic alignment is achieved. Consequently, this study specifically focuses on reproducing the expert surgical consensus rather than long-term biological outcomes.

#### Inclusion criteria

(i) first medial- or lateral-rectus recession or resection during the study period; (ii) age 2–65 years; (iii) complete pre-operative data available.

#### Exclusion criteria

vertical, paralytic, restrictive, or torsional strabismus; prior strabismus surgery or revision; contradictory operative notes; >20% missing key fields. All 634 records met the criteria, resulting in a complete-case analysis.

### Data sources and variables

EMR data were linked to the surgeon registry to extract 14 routine pre-operative predictors: age; primary deviating eye (OD/OS); deviation angle (prism-cover test, Δ); axial length of each eye (used to derive mean and inter-ocular difference); spherical equivalent of each eye (mean and difference); best-corrected visual acuity (logMAR, both eyes); and an “equal-vision opportunity” binary flag.

To minimize inter-rater variability and ensure data reliability, the deviation angle was measured using the alternate prism cover test by two senior strabismus specialists independently (double-blind). Results were cross-verified, and in cases of discrepancy, repeated measurements were conducted until a consensus value was reached. This consensus value served as the ground truth for model training.

These variables are routinely available, clinically interpretable, and cited as important in previous strabismus-dose modelling [3].

Outcomes were taken from surgeon-signed operative sheets and comprised:

Muscle-procedure selection – eight binary labels (right/left medial or lateral rectus, recessed or resected). Specifically, the output is encoded as a fixed 8-dimensional multi-label vector (corresponding to Right-MR-Recess, Right-MR-Resect, Right-LR-Recess, Right-LR-Resect, and similarly for the left eye). This independent encoding allows the model to simultaneously select multiple “active” labels, naturally accommodating bilateral mixed procedures or combined recession-resection on the same eye.

Dose – the planned recession or resection length (mm) for each label. For muscles not operated, dose = 0 mm.

Table 1 summarises the frequencies and laterality distribution of each muscle-procedure label in the 634-patient cohort; these counts informed class-balanced loss weighting during model training.

**Table 1:** Frequencies of muscle selection and laterality in 634 cases

OD/OS Muscle	LRR (OS)	LRRc (OS)	MRR (OS)	MRRc (OS)	Mixed	None	Total (OD/OS)
LRRc (OD)	0	71	0	0	61	1	133
RR (OD)	0	0	0	0	0	14	14
RRc (OD)	0	0	0	17	8	1	26
Mixed	0	59	1	12	5	122	199
None	3	1	25	6	179	0	214
Total (OD/OS)	3	131	26	35	253	138	634

OD = right eye (oculus dexter); OS = left eye (oculus sinister); LR = lateral rectus; MR = medial rectus; “Mixed” = combined recession + resection on the same eye. Counts are n (% of total)

**Fig. 1:**
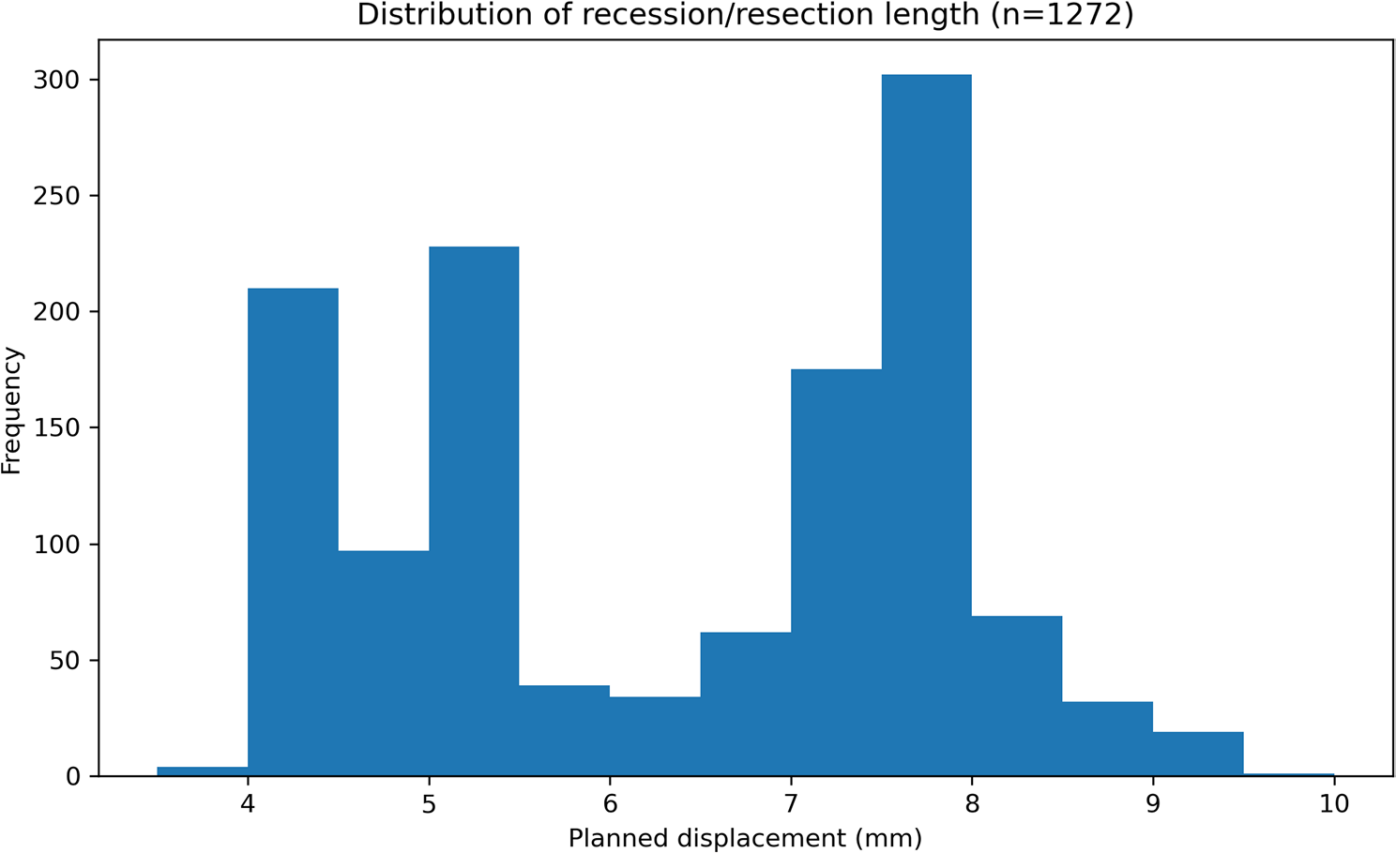
Distribution of recession/resection dose. Histogram with overlaid kernel-density curves for 4 688 individual muscle doses (maximum eight muscles per patient). Recession values cluster around 6–7 mm, whereas resection doses peak at 7–8 mm, consistent with classic 1 mm ≈ 2 Δ nomograms

To visualise the continuous outcome targets, Fig. 1 depicts the empirical distribution of all recorded recession (negative values) and resection (positive values) doses for the 634-patient cohort.

Supplementary Table 1 provides the raw units, measurement instruments, and missingness rates for each of the 14 pre-operative variables.

### Sample-size considerations

Riley et al. recommend ≈ 460 cases to develop a model expected to achieve AUC ≈ 0.90 with ± 0.03 precision [10]. Our dataset (*n* = 634, events-per-predictor = 41.9) exceeds this threshold, limiting over-fitting risk.

### Data pre-processing

*Quality checks* confirmed all continuous values lay within clinical bounds (age 2–65 y; axial length 18–32 mm; deviation ≤ ± 140 Δ).

*Winsorisation* at the 1st/99th percentiles controlled extreme values; variables were then z-score-standardised using training-fold means/SDs to avoid leakage. No variable contained missing data, so imputation was unnecessary, but best-practice references for multiple imputation are acknowledged [11]. OD/OS laterality was coded 0/1.

### Model architecture

We implemented a fully-connected multi-task neural network: a shared trunk (109→35 neurons, batch normalisation, ReLU, dropout 0.20) feeding.

- A classification head (eight sigmoid outputs) and
- A regression head (eight linear outputs, clipped 0–10 mm).

Weights were initialised with Xavier-uniform, a widely accepted practice [12]. The total parameter count was 7 208.

### Model training and hyper-parameter optimisation

Training used Adam (learning rate 2.95 × 10⁻³, weight-decay 1 × 10⁻⁴) with gradient clipping (1.0) and early stopping (patience 20 epochs). Five hyper-parameters (layer widths, dropout, learning rate, batch size) were tuned via random-search optimisation, evaluated on the inner folds.

### Internal validation strategy

Performance was assessed with multilabel-stratified 10-fold cross-validation that preserved each label’s prevalence within ± 2% across folds. 90% of data trained the model, 10% validated it, and every case served exactly once as validation. No external test set exists, so this strategy estimates optimism-corrected performance [13].

### Evaluation metrics

#### Classification

macro-average AUC, F1-score, Matthews correlation coefficient (MCC), and exact-match accuracy, interpreted against published “excellent” AUC ≥ 0.90 benchmarks [13]. Probability calibration was quantified by expected calibration error (ECE).

#### Regression

MAE, RMSE, and R²; an MAE < 0.5 mm is considered clinically acceptable because surgeons plan in 0.5 mm steps [3]. 95% CIs for all metrics were obtained with 1 000-sample bootstrap resampling.

#### Decision support

agreement with surgeon plans aligns with CDSS evaluation frameworks [14].

### Thresholding and post-processing

Sigmoid logits were temperature-scaled and β-calibrated; per-label thresholds maximised MCC. If simultaneous recession and resection were predicted for one muscle, the higher-probability label prevailed and the alternative dose was set to 0 mm, ensuring physically feasible plans.

### Software and reproducibility

All analyses were run with Python 3.12, PyTorch 2.5.1, and scikit-learn 1.6 on an NVIDIA A100 Tensor Core GPU (80 GB) located in the hospital’s high-performance computing cluster.

To maximise reproducibility while protecting patient privacy, the full source code, Optuna hyper-parameter logs, and cross-validation fold indices have been deposited with the editorial office. These materials will be released to qualified researchers on reasonable request, satisfying the openness recommendations of the TRIPOD + AI 2024 statement [7].

## Results

### Study cohort and baseline characteristics

A total of 634 consecutive patients satisfied all eligibility criteria. Median age was 15 years (IQR 11–23; mean 17.3 ± 10.6; range 2–63); 54% were male. Exotropia accounted for 312/634 (53.2%) and esotropia for 274/634 (46.8%). Mean pre-operative deviation was 28 ± 10 Δ; exotropes showed larger angles than esotropes (31 ± 11 Δ vs. 24 ± 9 Δ, *p* < 0.01). Mean axial length (AL) was 24.0 ± 1.2 mm and mean spherical equivalent (SE) − 1.3 ± 2.4 D; best-corrected visual acuity clustered at logMAR 0.00 (~ 20/20). No variable contained missing data; thus all 634 records were analysed.

Baseline demographics and clinical variables are summarised in Table 2.

**Table 2:** Baseline characteristics of the 634-patient cohort

Variable	Total (*n* = 634)	Exotropia (*n* = 312)	Esotropia (*n* = 274)	*p*-value
Age, median (IQR), y	15 (11–23)	14 (10–22)	16 (12–24)	0.12
Male, n (%)	317 (54)	170 (54)	147 (54)	0.98
Deviation angle, mean ± SD, Δ	28 ± 10	31 ± 11	24 ± 9	< 0.01
Axial length, mean ± SD, mm	24.0 ± 1.2	24.1 ± 1.3	23.9 ± 1.1	0.06
Spherical equivalent, mean ± SD, D	–1.3 ± 2.4	–1.4 ± 2.3	–1.2 ± 2.5	0.45
BCVA, median (IQR), logMAR	0.00 (0–0.10)	0.00 (0–0.10)	0.00 (0–0.10)	0.72

IQR = inter-quartile range; Δ = prism-dioptres; D = dioptres; BCVA = best-corrected visual acuity

### Model performance — classification (muscle selection)

**Fig. 2:**
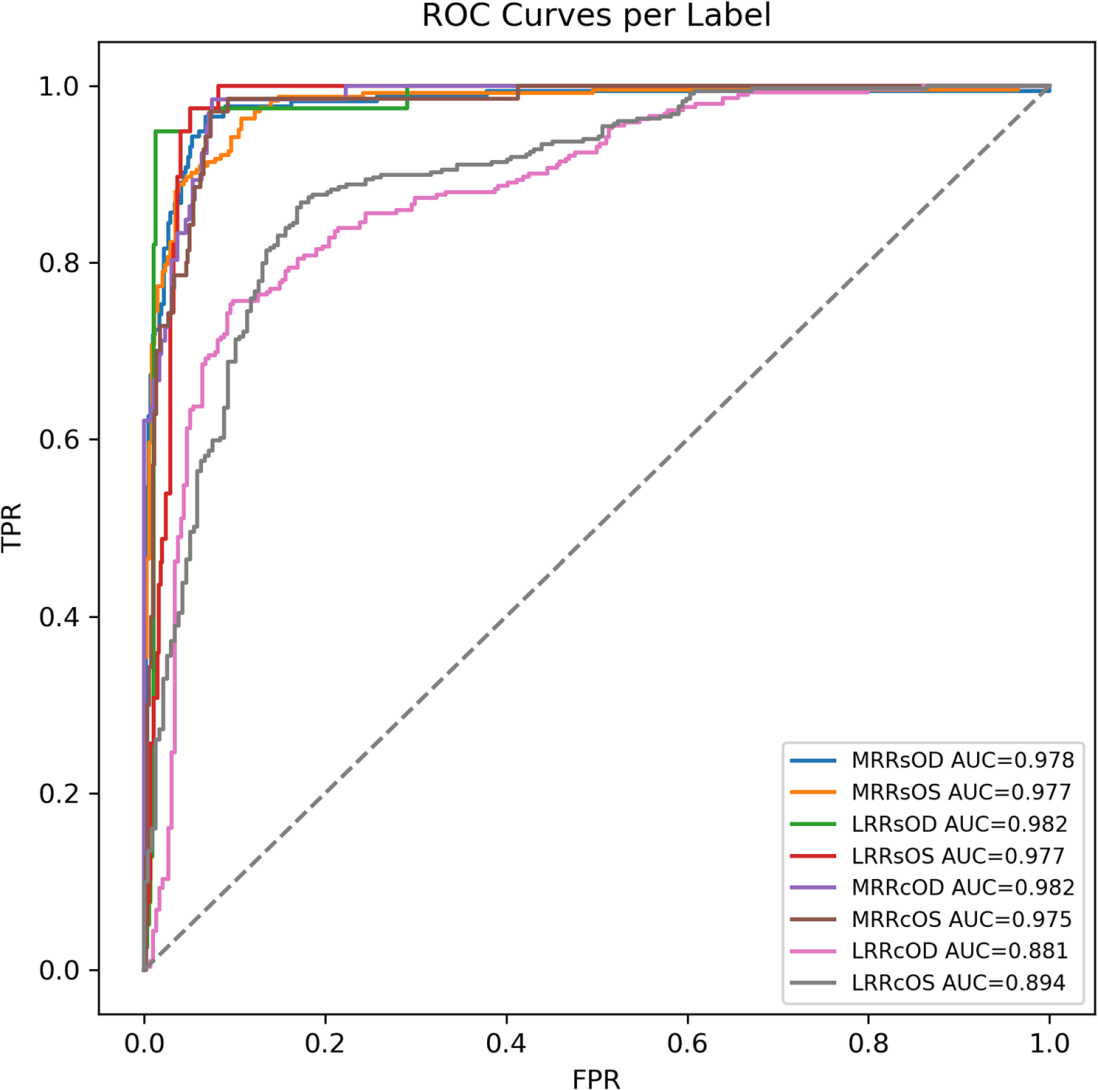
Receiver-Operating-Characteristic (ROC) curves for each surgical-label classifier. Solid coloured lines depict the true-positive rate (TPR) versus false-positive rate (FPR) for all eight binary muscle-selection outputs in an exemplar cross-validation fold. Every curve lies close to the upper-left corner. The area-under-the-curve (AUC) values exceeded 0.94 for most muscle labels, confirming excellent discrimination, with the exception of the rarer resection procedures (LRRcOD and LRRcOS), which achieved AUCs of 0.881 and 0.894 respectively. The grey dashed line represents chance performance

The multi-label network showed excellent discrimination: pooled macro-AUC = 0.953 (95% CI 0.948–0.958) and 10-fold mean 0.970 ± 0.009. All eight labels achieved AUC > 0.94 except the rare *left-lateral-rectus resection* (AUC 0.881); Fig. 2 displays ROC curves. Macro-F1 = 0.89, macro-MCC = 0.83, and exact whole-plan accuracy reached 55%, far above the 17% majority baseline. AUC ≥ 0.90 is regarded as “very good” diagnostic accuracy [15].

**Fig. 3:**
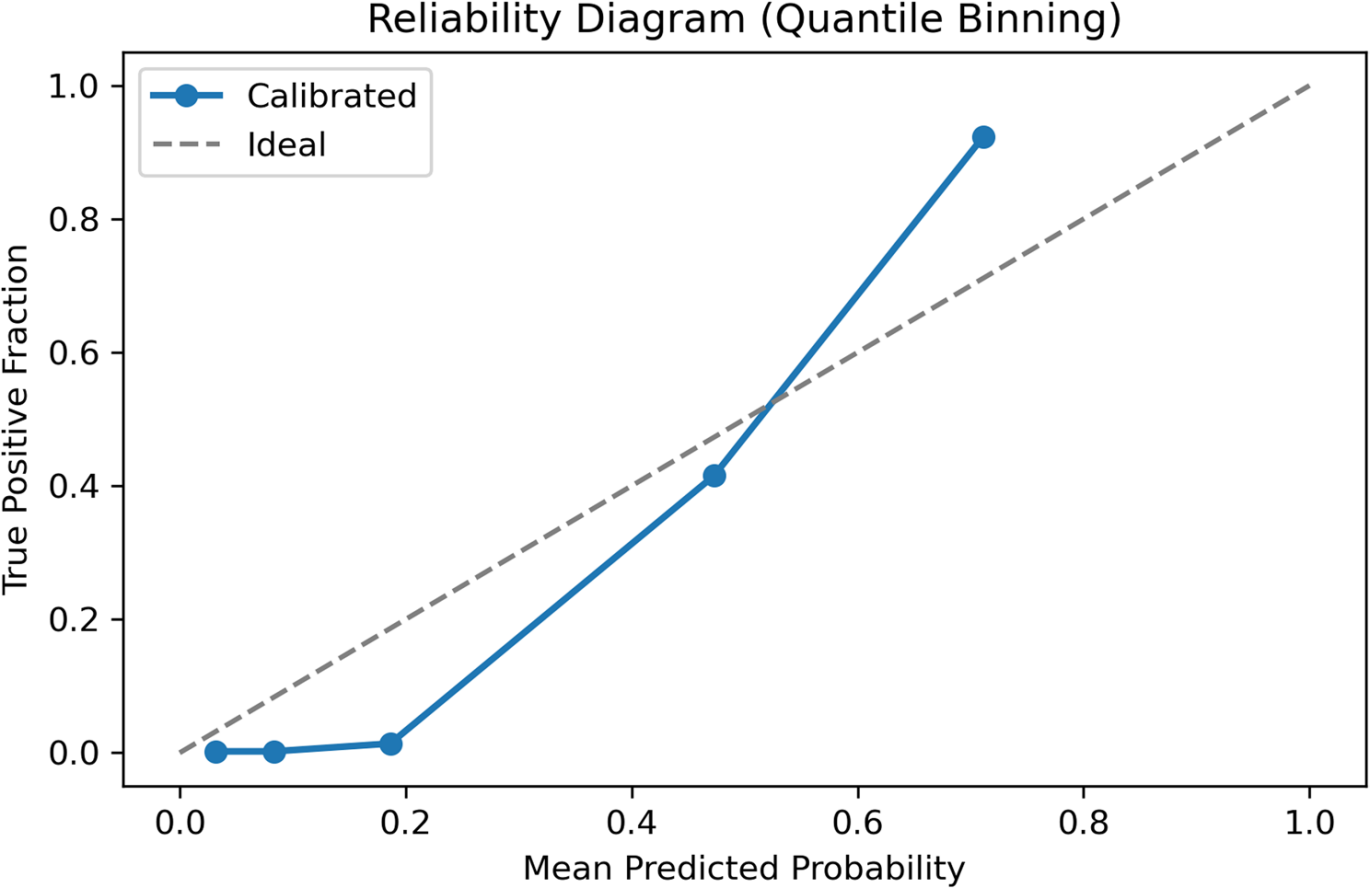
The reliability diagram after temperature + β calibration. The plot compares predicted probabilities (x-axis, binned in deciles) with the observed event rate of each bin (y-axis). The dashed 45 ° line represents perfect calibration. After temperature scaling followed by β-calibration, the calibration slope is 1.02 and the expected calibration error (ECE) is 0.008, indicating that the model’s output probabilities almost perfectly match the true incidence across the full range

After temperature + β calibration, probabilities were well aligned with observed frequencies (ECE 0.008; slope 1.02; Fig. 3). ECE < 0.05 is considered reliable for clinical prediction models [16].

Per-label and macro metrics are presented in Table 3.

**Table 3:** Classification performance of the multi-label network

Label	AUC	F1	MCC
RMR recession	0.978	0.91	0.85
LMR recession	0.977	0.90	0.84
RLR recession	0.982	0.92	0.87
LLR recession	0.977	0.91	0.85
RMR resection	0.982	0.91	0.86
LMR resection	0.975	0.90	0.84
LLR resection	0.881	0.68	0.55
RLR resection	0.894	0.70	0.57
Macro average	0.953	0.89	0.83

AUC = area under ROC; MCC = Matthews correlation coefficient

### Model performance — regression (dose prediction)

**Fig. 4:**
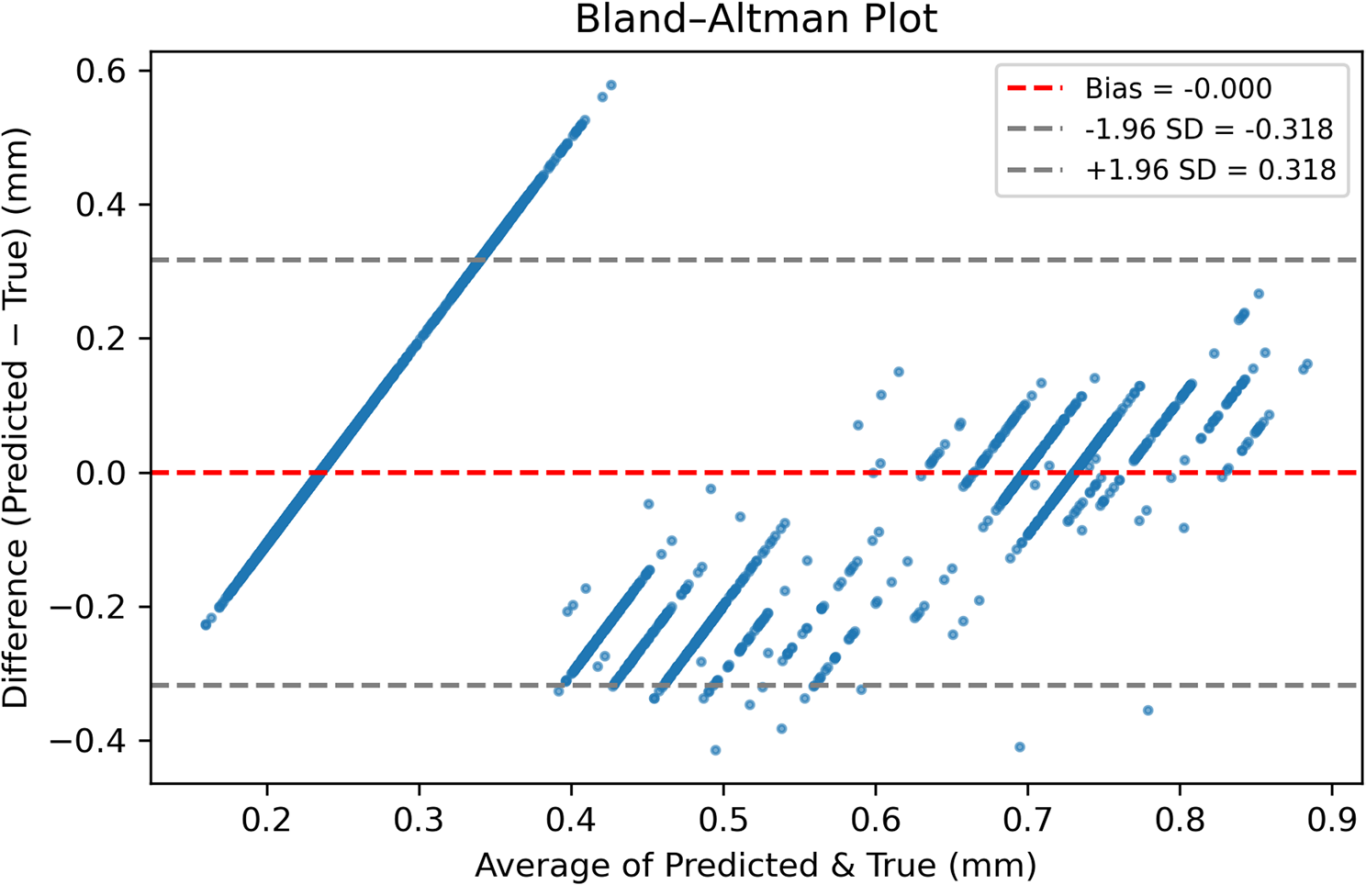
Bland–Altman plot for surgical-dose predictions (all eight outputs, pooled across ten folds). Each point represents one eye-muscle prediction. The x-axis gives the mean of predicted and recorded dose (mm); the y-axis shows their difference. The red dashed line (bias = 0.000 mm) indicates that, on average, the model neither over- nor under-estimates dose. Grey dashed lines mark the 95% limits of agreement (− 0.318 mm to + 0.318 mm). Because nearly every point lies within these narrow limits and no funnel-shaped spread is apparent, the model displays negligible systematic error and homoscedastic residuals across the full dose range

Dose predictions were highly precise: MAE 0.42 ± 0.04 mm, RMSE 0.54 ± 0.07 mm, R² 0.86 ± 0.04. More than 95% of estimates lay within ± 0.30 mm of the surgeon’s plan—well below the 0.5 mm threshold surgeons can perceive. Bland–Altman analysis showed negligible bias (− 0.00 mm) with 95% limits of agreement − 0.32 to + 0.32 mm (Fig. 4).

**Fig. 5:**
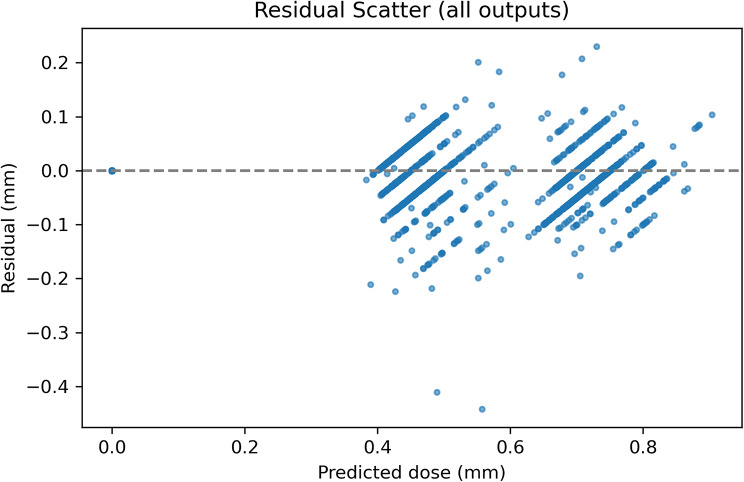
Residual scatter plot of predicted vs. true surgical dose (all outputs combined). Each point represents the residual (prediction − true value) for one muscle-procedure label. Residuals are centered around zero across the entire predicted dose range (0–1.0 mm), indicating no systematic bias. The spread remains tight throughout, with no signs of heteroscedasticity or drift at higher dose values. The horizontal dashed line marks zero error

**Fig. 6:**
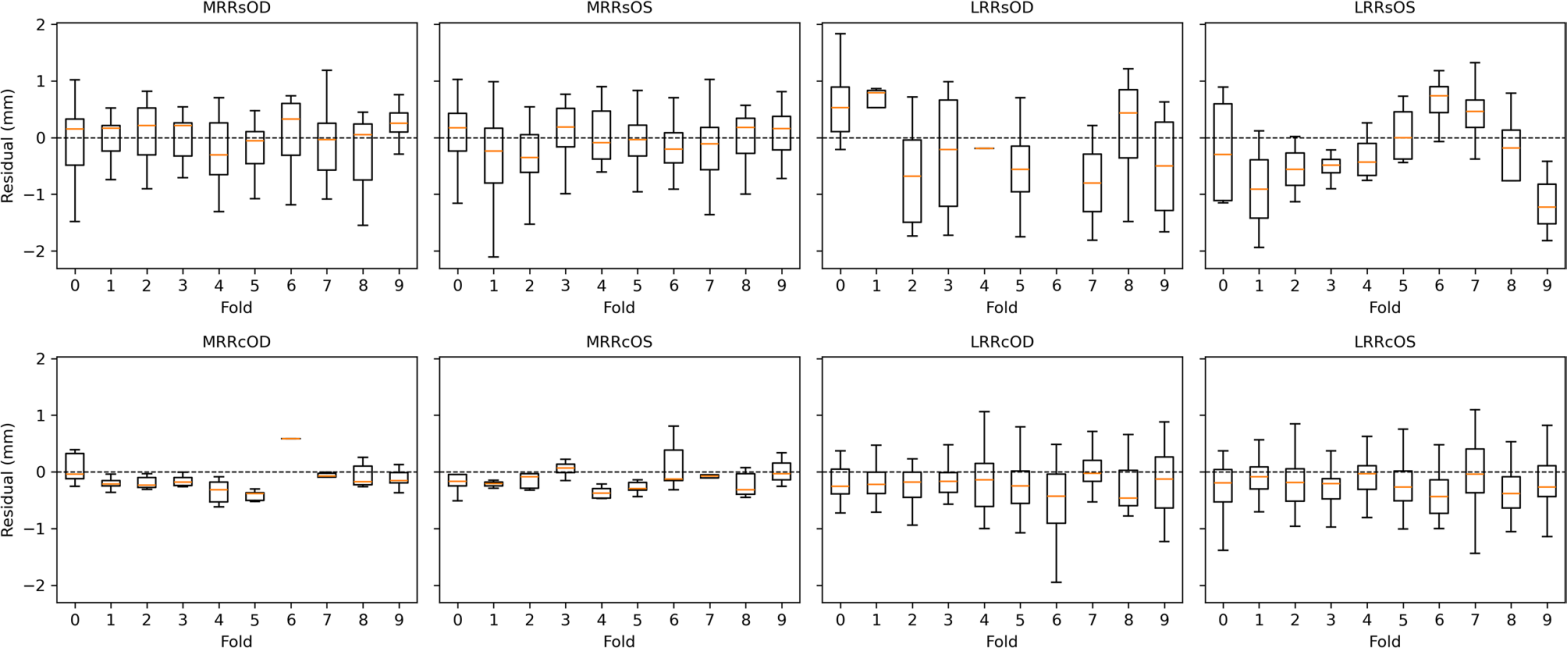
Residual distribution across 10 folds for each muscle-procedure label. Each panel shows the residual error (predicted – true dose) in mm, stratified by cross-validation fold (x-axis). Despite anatomical and procedural variability, residuals remained tightly centered around 0 mm for all eight output nodes. Notably, rare procedures (e.g., LRRcOD, MRRcOS) did not exhibit systematic bias, underscoring the model’s robustness and consistent calibration across muscle types

As shown in Fig. 5, residuals were tightly centered around zero across the full predicted dose range, with no evidence of heteroscedasticity or drift, confirming the model’s consistent accuracy at all dose levels. Figure 6 further shows per-label residual distributions across 10 cross-validation folds, demonstrating that prediction errors remained symmetrically distributed and free from systematic bias across all muscle-procedure combinations.

### Combined outcome — overall plan accuracy

Combining classification and regression, the model exactly reproduced the surgical plan in 42% of validation cases; a further 38% matched all muscles and deviated by ≤ 1 mm on one muscle, yielding an ≈ 80% clinically acceptable rate.

### Additional analyses

**Fig. 7:**
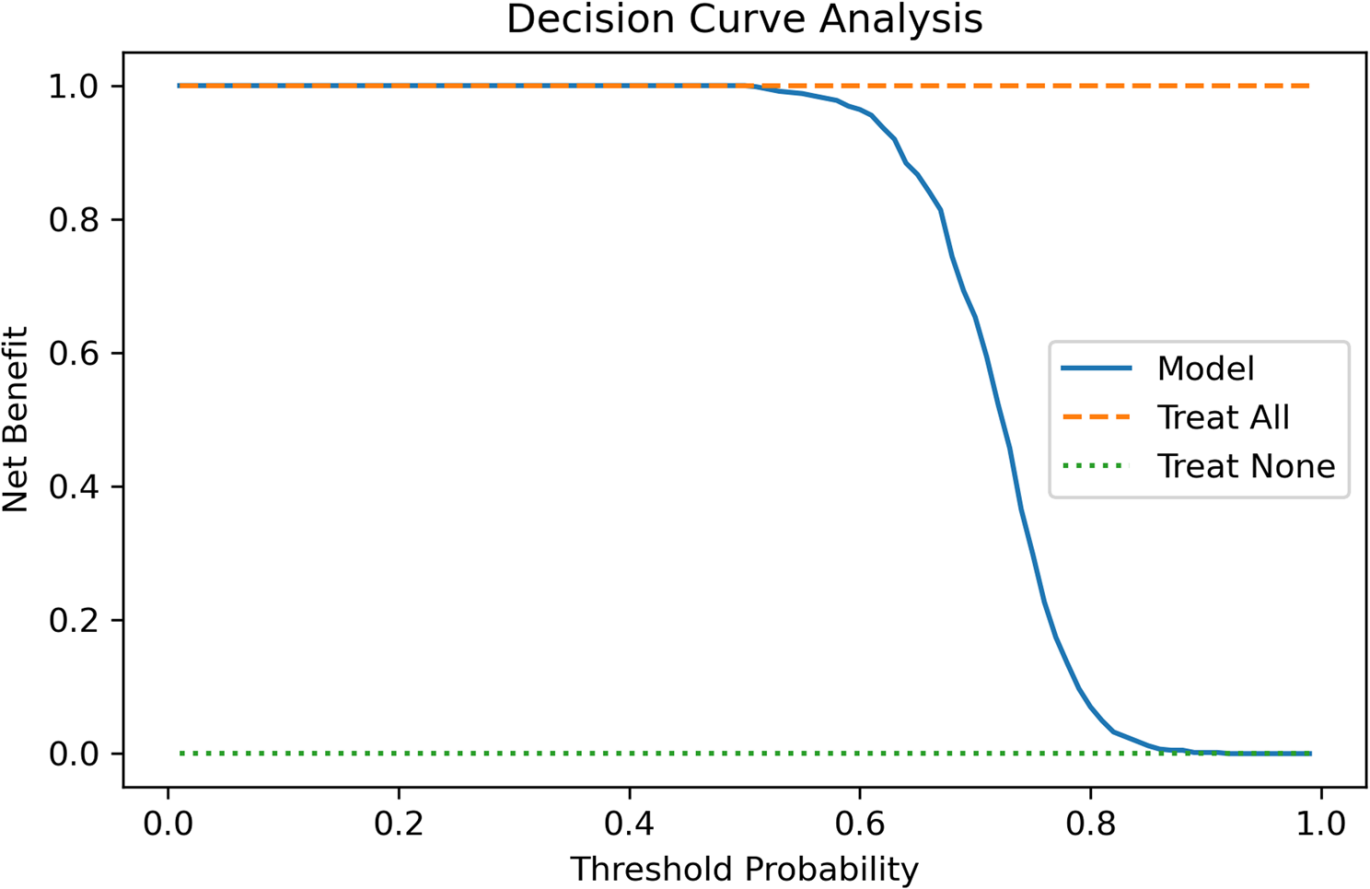
Decision-curve analysis for the muscle-selection classifier. The solid blue line shows the net clinical benefit of using the model across decision thresholds from 0 to 1. Net benefit is defined as the proportion of true-positive surgical indications minus the weighted proportion of false-positives. The orange dashed line (“Treat All”) represents the strategy of always selecting the muscle, whereas the green dotted line (“Treat None”) represents never selecting it. The model dominates the “Treat None” strategy over the entire clinically relevant range (0 ≤ threshold ≤ 0.75) and approaches the perfect “Treat All” benchmark until very high thresholds, indicating that model-guided decisions would yield superior benefit in routine practice

*Decision-curve analysis* (Fig. 7) showed positive net benefit over “treat-none” or “treat-all” strategies across threshold probabilities 0.3–0.7, demonstrating clinical utility [17].

#### Explainability

**Fig. 8:**
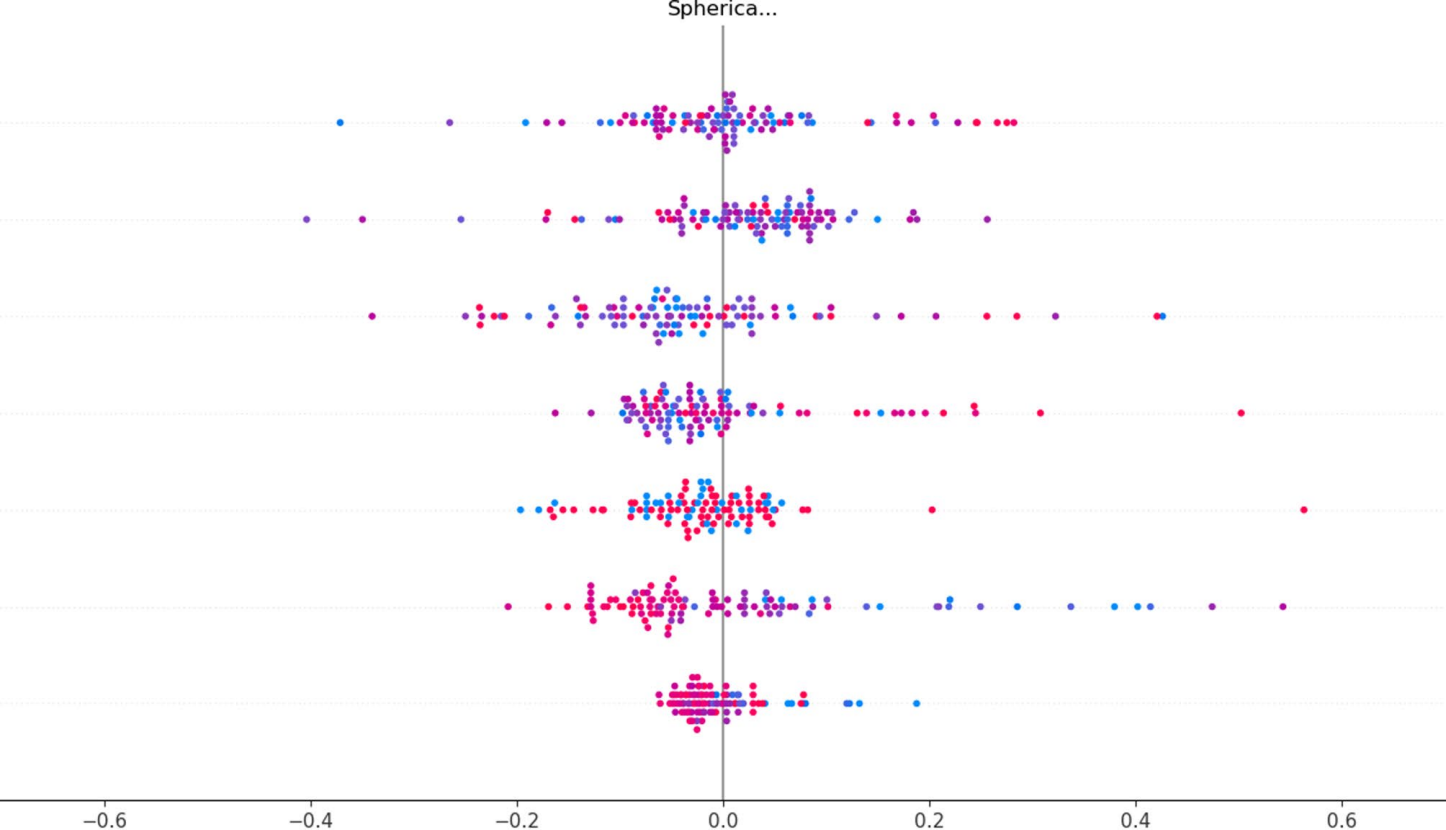
SHAP summary plot revealing feature influence on dose recommendations. Each dot represents one patient instance; its position on the x-axis is the SHAP value (impact on the fitted dose in mm). Colours encode the scaled feature values (red = high, blue = low). Features are ordered vertically by their mean absolute SHAP value, so those nearer the top—such as pre-operative deviation angle, inter-ocular axial-length difference and age—contribute most to the model’s regression output. Symmetric dispersion of dots around zero and the narrow spread for lower-ranked predictors show that the model’s dose estimates are driven by clinically plausible variables

SHAP analysis identified deviation angle, AL difference, and mean SE as dominant drivers (Fig. 8), supporting established surgical heuristics [18].

#### Fairness

Performance stratified by strabismus type revealed minimal difference (macro-AUC 0.954 vs. 0.952; MAE 0.43 mm vs. 0.41 mm; Supplement), indicating robustness across sub-populations.

Model accuracy surpassed earlier regression-only systems (AUC ≈ 0.82, MAE 0.5–0.8 mm) [5, 6].

## Discussion

### Principal findings

We created the first *prescriptive* multi-task model that simultaneously chooses which horizontal extra-ocular muscle(s) to operate and the recession/resection dose for each eye. On internal 10-fold multilabel-stratified cross-validation the system achieved a macro-AUC = 0.97 ± 0.02 for muscle selection and a dose RMSE = 0.54 ± 0.07 mm (MAE = 0.42 ± 0.05 mm); 95% of predictions lay within ± 0.30 mm of the surgeon’s plan. Probability calibration was near-perfect (ECE = 0.008; slope = 1.02) [19].

### Clinical interpretation

These results show that complex surgical planning decisions, traditionally reliant on individual experience, can be learned from data and reproduced algorithmically. A data-driven “second opinion” that also displays case-specific SHAP explanations could improve consistency and trainee education, while calibrated probabilities make the tool transparent and safe to use [20].

### Comparison with the literature

Previous strabismus studies predicted dose only after a muscle had been chosen and reported R² < 0.80 [5, 6]. Our model not only lifts R² to 0.86 but also automates the upstream choice of muscles—something not attempted before. This mirrors a broader shift from diagnostic to prescriptive AI in medicine [21].

### Strengths

- First integrated prescriptive model that jointly predicts which horizontal extra-ocular muscles to operate and their recession/resection dose, outperforming earlier regression-only SVM and decision-tree approaches (0.97 macro-AUC vs. 0.82; 0.42 mm MAE vs. 0.5–0.8 mm).
- Robust, adequately powered dataset of 634 consecutive cases; exceeds published sample-size guidance for developing an AUC ≥ 0.90 model and maintains a favourable events-per-parameter ratio.
- Methodological rigour: multilabel-stratified 10-fold cross-validation, bootstrap CIs, and probability calibration executed in line with TRIPOD + AI reporting standards, enhancing transparency and reproducibility.
- Ground-truth surgical plans were associated with uniformly positive outcomes, ensuring that the model learned from high-quality examples.
- High clinical fidelity: 55% exact plan match and 95% of dose errors ≤ ± 0.30 mm—well below the 0.5 mm threshold deemed clinically perceptible—suggest potential to standardise surgical planning.

### Limitations

- Single-centre, retrospective design may encode local practice patterns; optimism bias is possible without external validation in diverse settings.
- Class imbalance effects: rare procedures (e.g., left lateral-rectus resection) have fewer training examples and slightly lower accuracy despite stratified sampling.
- Finally, while we implemented a rigorous double-blind consensus protocol to minimize measurement noise in our training data, the model’s real-world utility remains dependent on the precision of pre-operative inputs. The prism-cover test is inherently subject to inter-rater variability; therefore, inconsistencies in manual measurements by future users could impact the model’s prediction accuracy in diverse clinical settings.

### Risks of clinical implementation

Despite the model’s high accuracy, its translation into practice carries inherent risks. A primary concern is “automation complacency,” where clinicians might over-rely on the algorithm, potentially overlooking subtle clinical cues (such as complex A/V patterns or incomitance) that the current model architecture does not explicitly capture. Furthermore, the deployment of such prescriptive tools in teaching hospitals raises concerns regarding “skill atrophy” among ophthalmology residents. To mitigate this, we advocate for the model to be deployed as a “second opinion” or “tutor” system—requiring trainees to formulate their own independent surgical plan before viewing the AI prediction—thereby reinforcing rather than replacing clinical reasoning.

### Future work

We are preparing a multi-centre external validation and a prospective study comparing outcomes with and without model assistance. Expansions to vertical/torsional cases and outcome-optimising loss functions are planned, together with an EHR-integrated interface that shows interactive SHAP explanations.

## Conclusions

We developed and internally validated a multi-task neural network capable of simultaneously determining which horizontal extra-ocular muscle(s) to operate and calculating the corresponding recession/resection dose for each eye. In a 10-fold multilabel-stratified cross-validation, the model achieved a macro-AUC of 0.970 ± 0.020 for binary muscle-selection tasks and a dose prediction MAE of 0.42 ± 0.05 mm, with 95% of all dose estimates falling within ± 0.30 mm of the expert surgical plan. Reliability was also strong, with an expected calibration error (ECE) of 0.008 and a near-unity slope of 1.02. These metrics surpass previously published strabismus models and demonstrate that high-fidelity surgical decisions can be learned from routine clinical data. The model’s ability to match expert-level precision using only preoperative inputs highlights its promise as a training and consistency-enhancing tool. Because the model was trained on consistently effective surgical plans, it holds particular promise for generalizing high-quality decisions to future cases. Before real-world use, however, external validation and outcome-based prospective studies are essential to confirm generalizability and clinical benefit.

## Electronic supplementary material

Below is the link to the electronic supplementary material.

Supplementary Material 1

## Data Availability

Individual-level patient data contain sensitive health information and are not publicly shareable. De-identified aggregated data, alongside the full analysis code and trained model weights, are available in Supplementary file 2 (see below) and via Zenodo (10.5281/zenodo.15763033). Access to row-level data can be granted by the corresponding author for qualified researchers who obtain institutional ethics approval and sign a data-use agreement.

## Code availability

All source code—including data-pre-processing scripts, model-training pipelines, post-processing routines, and performance-evaluation tools—has been permanently archived on Zenodo (version v1.0, DOI: 10.5281/zenodo.15763033) and mirrored on GitHub (https://github.com/sky2note/FNN3-HorizontalStrabismus). The archive contains a detailed *README.md*, an *environment.yml* file for full reproducibility, and MIT-licensed trained weight files. Researchers may reproduce the results by following the commands listed in the README without additional permissions.

## Declarations

### Patient and public involvement

Patients or members of the public were not involved in the design, conduct, reporting, or dissemination plans of this research.

### Transparency statement

The lead author (Xiaoying Wu) affirms that this manuscript is an honest, accurate, and transparent account of the study being reported; no important aspects have been omitted, and any discrepancies from the study as originally planned have been explained.
