# Supplementary material for: Development and internal validation of a prescriptive multi-task learning model for horizontal strabismus surgery planning
Source: BMC Ophthalmol. 2026 Jan 21;26:37. doi: 10.1186/s12886-026-04628-9 (PMC12828921; doi:10.1186/s12886-026-04628-9)
Supplement: Supplementary file 1 — Supplementary Material 1 [file 12886_2026_4628_MOESM1_ESM.docx]

# Supplementary Materials

## Supplementary Table

Supplementary Table 1: Raw-unit definitions, measurement instruments and missing data.

| Variable (unit) | Mean ± SD (raw) | Missing n (%) | Measurement instrument / timing | Variable (unit) |
| --- | --- | --- | --- | --- |
| Age (years) | 17.25 ± 10.58 | 0 | Electronic medical record; extracted at final pre-op visit | Age (years) |
| Prism deviation (Δ) | –37.43 ± 41.87 | 0 | Good-Lite horizontal prism bar (1–45 Δ); distance fixation, photopic light | Prism deviation (Δ) |
| Axial length OD (mm) | 24.12 ± 1.41 | 0 | IOLMaster 700; swept-source OCT | Axial length OD (mm) |
| Axial length OS (mm) | 24.07 ± 1.50 | 0 | IOLMaster 700 | Axial length OS (mm) |
| Spherical equivalent OD (D) | –1.41 ± 2.57 | 0 | Topcon KR-800 autorefractor | Spherical equivalent OD (D) |
| Spherical equivalent OS (D) | –1.27 ± 4.39 | 0 | Topcon KR-800 | Spherical equivalent OS (D) |
| Corrected VA OD (decimal) | 1.14 ± 0.25 | 0 | Sloan ETDRS chart (4 m, ISO 8596) | Corrected VA OD (decimal) |
| Corrected VA OS (decimal) | 1.10 ± 0.31 | 0 | Sloan ETDRS chart | Corrected VA OS (decimal) |
| AxL_mean (mm) | 24.10 ± 1.44 | 0 | Derived: (Axial length OD + OS)/2 | AxL_mean (mm) |
| AxL_diff (mm) | 0.05 ± 0.38 | 0 | Derived: | AxL_diff (mm) |
| SphEq_mean (D) | –1.34 ± 3.48 | 0 | Derived: (SE OD + OS)/2 | SphEq_mean (D) |
| SphEq_diff (D) | 0.14 ± 3.08 | 0 | Derived: | SphEq_diff (D) |
| Primary deviating eye | OS 344 (58.7 %); OD 242 (41.3 %) | 0 | Clinical assessment, prism-cover test | Primary deviating eye |
| Equal-Vision Opportunity | 114 Yes (19.5 %); 472 No (80.5 %) | 0 | Binary flag; defined per local guidelines | Equal-Vision Opportunity |

This table provides raw-unit definitions, measurement protocols, and missing data rates for all 14 pre-operative input variables used in the model. All measurements were extracted from the electronic medical record or standard ophthalmic instruments. Derived variables were calculated directly from recorded values (e.g., mean axial length). No variable had missing data, confirming a complete-case analysis.

## Supplementary file

| File | Description |
| --- | --- |
| 1 | TRIPOD-AI reporting checklist (.pdf) |
| 2 | Source code, trained weights and aggregated dataset for reproduction (.zip, 3.6 MB; Zenodo DOI: 10.5281/zenodo.15763033) |
